# Selective transfer of maternal antibodies in preterm and fullterm children

**DOI:** 10.1038/s41598-022-18973-4

**Published:** 2022-09-02

**Authors:** Sepideh Dolatshahi, Audrey L. Butler, Christian Pou, Ewa Henckel, Anna Karin Bernhardsson, Anna Gustafsson, Kajsa Bohlin, Sally A. Shin, Douglas A. Lauffenburger, Petter Brodin, Galit Alter

**Affiliations:** 1 Ragon Institute of MGH, MIT and Harvard, Cambridge, MA USA; 2 Biomedical Engineering Department, University of Virginia, Charlottesville, VA USA; 3 Science for Life Laboratory, Department of Women’s and Children’s Health, Karolinska Institutet, Stockholm, Sweden; 4 Department of Clinical Science, Intervention and Technology, Karolinska Institutet, Stockholm, Sweden; 5 Karolinska University Hospital, Stockholm, Sweden; 6 Department of Biological Engineering and Center for Gynepathology Research, Massachusetts Institute of Technology, Cambridge, MA USA

**Keywords:** Infectious diseases, Innate immunity, Systems biology

## Abstract

Preterm newborns are more likely to suffer from infectious diseases at birth compared to children delivered at term. Whether this is due to compromised cellular, humoral, or organ-specific development remains unclear. To begin to define whether maternal–fetal antibody transfer profiles differ across preterm (PT) and fullterm (FT) infants, the overall quantity and functional quality of an array of 24 vaccine-, endemic pathogen-, and common antigen-specific antibodies were assessed across a cohort of 11 PT and 12 term-delivered maternal:infant pairs from birth through week 12. While total IgG levels to influenza, pneumo, measles, rubella, EBV, and RSV were higher in FT newborns, selective Fc-receptor binding antibodies was noted in PT newborns. In fact, near equivalent antibody-effector functions were observed across PT and FT infants, despite significant quantitative differences in transferred antibody levels. Moreover, temporal transfer analysis revealed the selective early transfer of FcRn, FcγR2, and FcγR3 binding antibodies, pointing to differential placental sieving mechanisms across gestation. These data point to selectivity in placental transfer at distinct gestational ages, to ensure that children are endowed with the most robust humoral immunity even if born preterm.

## Introduction

In the US alone, more than 10% of babies are born at less than 37 weeks of gestation, with 2.75% born at less than 34 weeks of gestation^1^. Babies born earlier than 39 weeks have a greater incidence of respiratory complications, low blood sugar, feeding problems, as well as an elevated risk of developing diseases over time^2,3^. While rates of preterm (PT) births are declining in the developed world, PT births are on the rise in the developing world^4^, with 15 million PT births world-wide each year^5^, with premature birth representing the second leading cause of death among children under 5 years of age across the globe^6^.

In the last weeks of pregnancy, significant development of the brain, lungs, liver, and skin occur in parallel to significant weight gain, that collectively ensure the survival of the neonate outside the uterus. Significant changes also occur in immune cellular maturation and function, in the last weeks of gestation and first months of life, pointing to a rapid evolution of the immune system in preparation for exposure to the non-sterile world^7,8^. However, even when fully developed, neonates are born with a largely naïve immune system, that must quickly adapt to the new environment and prevent infections^7,8^. To help their offspring, mothers actively transfer copious levels of IgG antibodies to infants in utero, to passively immunize the neonates against pathogens previously encountered by the mother. Thus, the final months/weeks of gestation are critical for final developmental events and to fully immunologically arm the neonate to prepare for the non-sterile life outside utero.

Commonly attributed to developmental and immune prematurity, PT babies are more susceptible to infections^2^. This includes infections in the lungs, skin, kidneys, eyes, bladder, and gastrointestinal tract. Specifically, higher susceptibility in PT infants has been reported for Varicella-Zoster virus^9^ and viral respiratory infections^2^ even when transferred antibody levels are comparable. However, whether this increased susceptibility of PT children is related to an impairment in the immune system or due to incomplete transfer of protective antibodies from the mother to neonate remains unclear. If the latter is true, vaccine strategies could be developed for pregnant women to induce enhanced transfer of antibodies to infants against pathogens that disproportionately affect PT neonates.

Maternal immunity is largely transferred across the placenta in the form of IgGs, actively captured from the maternal circulation and transferred in a neonatal Fc-receptor (FcRn) dependent manner to the infant^10–13^. Placenta transfer occurs throughout pregnancy, with the highest levels of antibodies transferred in the third trimester^14^. PT infants have been shown to possess lower antigen-specific IgG levels than FT infants^15^. However, despite the lesser quantities of antibodies in PT infants, a recent high-dimensional analysis of the transferred antibody repertoire found comparable antibody repertoires and even comparable neutralizing activity in PT compared to term infants, arguing for selective transfer of particularly functional antibodies early in pregnancy^16^. These data challenge the dogma of non-specific antibody transfer and suggests that the placenta may selectively transfer functionally enhanced antibodies earlier in pregnancy. However, whether functional antibodies, able to bind differentially to Fc receptors and activate innate immune cell functions, are transferred differentially across PT and FT infants remains unclear, but could provide clues related to immune vulnerabilities.

Early models suggested that placental antibody transfer was largely an unbiased process mainly driven by FcRn affinity for different IgG subclasses (IgG1 > IgG4 > IgG3 > IgG2), indiscriminately transferring any antigen-specific antibody to the neonate^10,17,18^, but more recent data point to the preferential transfer of unique antibody specificities^19,20^, that may be driven via Fc-glycan preferences of FcRn and/or other collaborating Fc-receptors expressed within the placenta^21^. However, whether placental Fc-preferential transfer occurs constantly throughout pregnancy or evolves over time to select more functional antibodies and empower neonates with differential immunity, should they be born early, remains unclear. Thus, here we comprehensively profiled and compared the Fc-binding profiles of antibodies in mothers, cord blood and postnatal samples from children born at different gestational ages across 24 pathogen/antigen-specific antibody populations.

### Ethical approval

Informed consent was obtained from all participants, and for neonates from a parent and/or legal guardian. All experimental protocols were approved by Massachusetts General Hospital Institutional Review Board. All methods were carried out in accordance with relevant guidelines and regulations.

## Results

### Cohort characteristics

21 mother/child pairs, including 11 extremely preterm (PT) and 12 fullterm (FT) sets were included in this study. To eliminate environmental confounders, only samples collected at the Karolinska University Hospital, Huddinge, were included in this analysis^8^. Samples included cord blood and peripheral blood plasma from 1 week, 4 weeks, and 12 weeks post birth. Maternal peripheral blood plasma samples were also collected at birth. Gestational ages for PT newborns ranged from 24 to 29 weeks and FT from 37 to 40 weeks. Gestational age was correlated with birth weight between the two groups (Table 1). Other characteristics such as maternal age, delivery mode, male–female proportion, breastfeeding status, maternal Body Mass Index (BMI), primipara, and smoking status (Table 1) and infant vaccination status (Table 2) were comparable between the PT and FT groups. Additionally, the cohort was matched for the timing of seasonal maternal influenza immunization across the pre- and FT infants, with matched seasonal recruitment within the PT and FT dyads. Plasma was purified from all maternal and cord samples using the same protocol from sodium-heparin tubes.

**Table 1:** Cohort characteristics.

Group	Preterm, n = 11	Term, n = 12
Birth weight, grams	1104 ± 226 (601–1398)	3655 ± 509 (3055–4432)
Gestational age, week^+days^	27^+6^ (24^+2^–29^+3^)	39^+3^ (37^+0^–41^+6^)
Male/female (% male)	4/7 (36)	5/7 (42)
Vaginal delivery (%)	6 (55)	8 (67)
Maternal age, years*	32 ± 6 (24–43)	34 ± 6 (26–44)
Maternal Body Mass Index, BMI	24 (18–26)*	25 ( 21–30)*
Primipara	10/11 (90)	7/12 (58)
Caucasian (%)	7/11 (64)*	10/12 (83)
Smoking/tobacco (%)	0/11 (0)*	3/12 (25)
Education at university level (%)^#^	6/11 (55)*	8/12 (67)

*Mean ± SD (minimum–maximum) if nothing else mentioned.

^#^Data missing n = 1.

^#^All mothers had at least 10–12 years of school.

**Table 2:** Sample characteristics.

	Preterm	Term
**Mother sample, n = 43**		
Week 1	11	12
Umbilical cord	7	11
**Baby sample, n = 53**		
Day 0–1 (obtained hours after birth)	2	0
Week 1	9	9
Week 4	10	0
Week 12	11	12
**Children vaccinated before any sample (%)**	9 (82)	9 (75)
Rotatec/rotarix^a^	1	7
Prevenar 13^b^	8	9
Infanrix hexa^c^	9	9
Synagis^d^	1	0
Varicellon^e^	1	0

^a^Rota virus.

^b^Pneumococci.

^c^Diphteria, tetanus, pertussis, hep B, polio, Hib.

^d^Palivizumab, immunoglobulins for respiratory synthycial virus.

^e^Immunoglobulin for Varicella-zoster virus.

### Fc-evolution in early life

**Figure 1:**
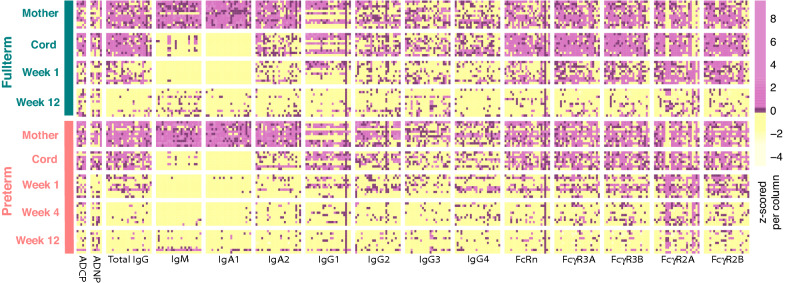
The evolution of the humoral response early in life. The heatmap depicts antigen-specific antibody measurements including antibody-dependent cellular phagocytosis (ADCP, for 5 antigens), antibody-mediated neutrophil phagocytosis (ADNP, for 6 antigens), total IgG, IgM, IgA1-2, IgG1-4, FcRn, FcgR3A, 3B, 2A, and 2B levels across 24 antigens (“Methods”, Table S1). Each column represents an antigen-specific measurement, always plotted in the same order for each Fc-measurement except for ADCP and ADNP, i.e. measles, mumps, rubella, pertussis toxin, influenza, VZV, polio, HepA, pneumo, Adeno T5, Adeno T40, Norovirus, Parvo VP2, tetanus, diphtheria, HSV1, HSV2, CMV, EBV, RSV, Human Histone H3, nArah2, nBosd8, nBetv1. Antigen specificities from left to right for ACDP: RSV, pneumo, pertussis toxin, rubella, influenza. Antigen-specificities for ADNP from left to right: rubella, pertussis toxin, influenza, pneumo, CMV, RSV. Each data type was normalized to have a zero mean and unit variance. Pink, black, and yellow depict high, mean, and low levels, respectively.

Transferred IgG increases with the trimesters^14^, resulting in highest levels of antibody transfer in the third trimester of gestation^10,17,18^. Consequently, PT infants are believed to have a compromised immune response due to lower levels of antibody transfer^22,23^. Given our emerging understanding for potential selective transfer of antibodies across the placenta, we comprehensively profiled the humoral immune response across 24 vaccine or pathogen-derived antigens (Table S1) in a cohort of 11 PT and 12 FT maternal:child pairs over the course of the first 3 months of life (Fig. 1)^8^.

IgGs, IgM, IgAs, and Fc-receptor binding antibodies were highly heterogeneous among mothers of both PT and FT babies (Fig. 1). However, expected patterns were observed across these isotypes over time. Specifically, IgG and IgG subclasses were detectable at week 0 in the cords, and then declined by week 4 and 12 in peripheral blood as the antibodies waned. Conversely, substantial levels of IgM and IgA1 responses were only detected in the maternal plasma, were absent in the cord, and IgM and IgA1 began to evolve in a limited number of infants by 3 months following birth. Interestingly, Fc-receptor binding antibodies were detectable in the mothers, were detectable in the cords at birth, but then decayed rapidly to very low levels by the third month of life, with a more rapid and profound decline in the PT infants. Similar profiles were detected for antigen-specific antibody dependent cellular phagocytosis (monocyte phagocytosis, ADCP) and antibody dependent neutrophil phagocytosis (ADNP), with variable but detectable functions detected across antigens in the mothers as well as the cord. However, the decline in transferred functional antibodies was similar across the PT and FT baby sets, with some novel ADCP responses appearing to particular antigens by 3 months of life.

Despite the similarities in transfer and decay across the maternal:infant groups, a correlation matrix of all antibody features between mother and cord hinted at differential coordination of antibody transfer to PT and FT infants (Fig. S1A,B). Specifically, while antibody profiles were more highly coordinated in FT maternal:cord pairs, antibody transfer relationships were less coordinated in PT dyads. Of note, the pneumo- and pertussis-specific antibody features were positively correlated in term maternal:cord pairs, but were not correlated in the PT pairs. Similarly, correlations were largely negative (not statistically significant) in PT pairs. Thus collectively, these data highlight less coordinated antibody transfer profiles in preterm infant pairs decay profiles across PT and FT dyads.

### Convergence of antibody profiles over time

**Figure 2:**
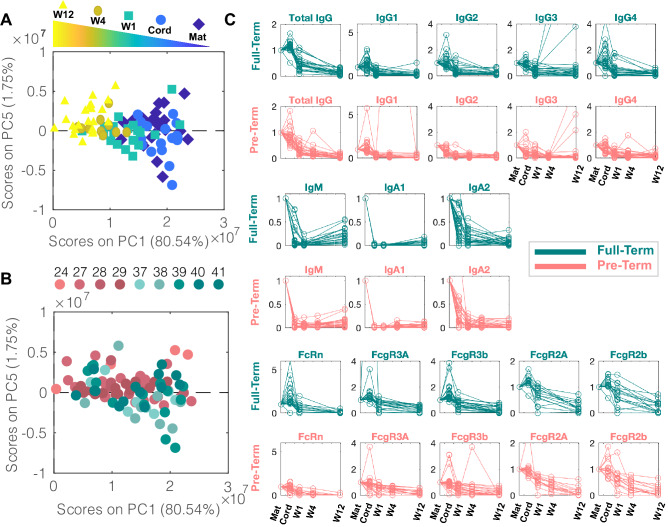
Convergence of antibody profiles over time. (**A**, **B**) Principal Component Analysis (PCA) was performed to reduce the dimensions of data from 323 to 5 dimensions to visualize the major axes of variation across the whole dataset. Each dot represents a sample. (**A**) 80% of the variance was captured by PC1, capturing the major variance in the data, picking up the evolution of the humoral profiles over time. The dot color represents the sample origin (maternal, cord, and time after birth). (**B**) In contrast to PC1, PC5 captures differences in antibody profiles across the PT and FT infants, where the dot color depicts the gestational group of the sample. (**C**) Each trendline represents the median of each antigen-specific measurement. These were normalized to the median of the maternal level of that antigen-specific measurement such that all trendlines start at a normalized value of 1. These were calculated separately for PT (pink) and FT (green) infants. One trendline is included per antigen per graph. Median tetanaus-, diphtheria-, and pertussis-specific (tdap) IgG3 peak at W12 for both pre-term and FT children, consistent with the Tdap administration to children between 6 and 8 weeks in Sweden.

Accumulating data point to striking differences in cellular immune function and frequencies in neonates compared to their mothers’, irrespective of gestational age, that are lost over the first few months of life^8^. To begin to examine whether similar patterns may exist within the humoral immune response, we used an unsupervised principal component analysis (PCA) to examine overall antibody profiles across the timepoints and samples (Fig. 2A). Separation was observed across timepoints, where mothers and cord samples scattered together, but a progressive shift was observed for infant antibody profiles with time from birth (Fig. 2A). Moreover, while the level of variation in maternal:cord samples was significant, less variation was observed in antibody profiles at weeks 1–4, with renewed variation appearing at 3 months following birth.

Interestingly, to determine whether this unsupervised approach could pick up any variation between FT and PT babies, each principal component (PC) was examined systematically. PC1 picked up variation within groups (Fig. 2A). Conversely, PC5 showed interesting partial discriminatory power between PT (pink) and term (green) infants (Fig. 2B). PC2-PC4 captured variation in the data that was not related to groups or PT and FT status (Supplemental Fig. 2A–C). Interestingly, the greatest variation in antibody profiles across the FT and PT infants occurred at birth, and these differences were lost overtime. Thus, collectively, these data highlight the differences in PT infant immunity at birth, that wanes in both groups and converges during the first few weeks of life.

To further gain a sense of what contributes to this variation between the two groups of infants, we next examined differences in the transfer and kinetics of decay of antibody subclass, isotype, and Fc-receptor binding profiles over time (Fig. 2C). As expected, evidence of preferential transfer of IgGs was observed in the FT babies compared to IgA and IgM transfer. Conversely, less selective transfer was observed among the IgGs in premature infants, albeit IgAs and IgMs were also deliberately not transferred. As expected, IgG transfer was linked to enhanced FcRn binding in FT infants after birth, coincident with a small, but heterogeneous, increase in FCγR3 binding antibodies and more persistent FCγR2 binding antibodies in the FT infants. Importantly, the robust increase in FcRn binding in FT infants was disproportionate to the level of transferred IgG1 levels, pointing to a qualitative shift in antibody quality selectively trapped and transferred to infants at birth. In contrast, PT infants exhibited a diminished FcRn binding signature, and more limited FCγR3 binding antibody levels right after birth, likely related to diminished IgG1 transfer, but the trends of maternal to cord levels were more similar across the groups for FCγR2 binding (Fig. 2C, last two rows). These data highlight expected antibody isotype subclass differences across FT and PT infants linked to unexpected Fc-receptor binding profiles over time.

### Selective impaired transfer of particular antigen-specific antibodies in PT children

**Figure 3:**
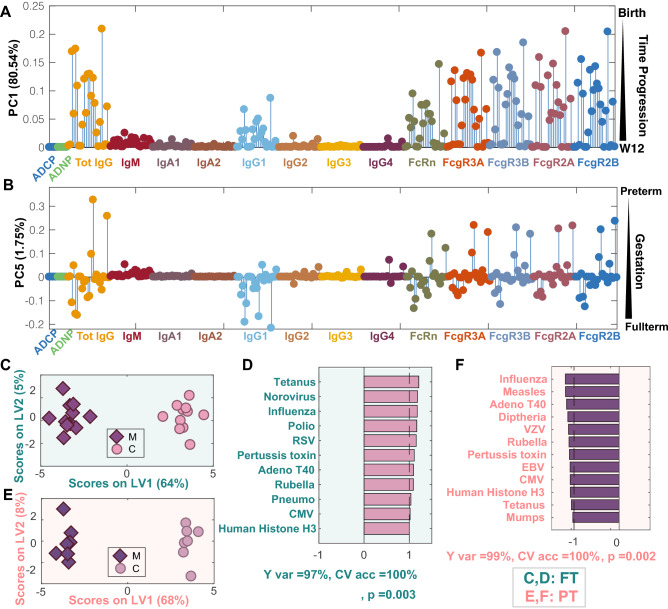
Antigen-specific transfer varies in PT infants. (**A**, **B**) The graphs depict the loading values on PC1 (**A**) and PC5 (**B**) of the PCA scores plot from Fig. 2. (**A**) Loadings on PC1 capture the changes over time. Each dot represents an antigen, and dot colors depict different Fc-readouts. (**B**) Loadings on PC5 reflect differences with gestational age. The order of antigens shown from left to right for all Fc measurements with the exception of ADCP and ADNP are: measles, mumps, rubella, pertussis toxin, influenza, VZV, polio, HepA, pneumo, Adeno T5, Adeno T40, Norovirus, Parvo VP2, tetanus, diphtheria, HSV1, HSV2, CMV, EBV, RSV, Human Histone H3, nArah2, nBosd8, nBetv1. Antigen specificities from left to right for ACDP: RSV, pneumo, pertussis toxin, rubella, influenza. Antigen-specificities for ADNP from left to right: rubella, pertussis toxin, influenza, pneumo, CMV, RSV. (**C**) ML-PLSDA depicts the antigen-specific differences across FT maternal:cord pairs. The model was orthogonalized such that the separation of maternal and cord antibody profiles was captured on Latent Variable 1 (LV1), capturing 83% of the Y-variation. Conversely, LV2 captures the variability in the antibody features that do not contribute to the separation (Y var = 0%). (**D**) The bar graph depicts the VIP scores for loadings on LV1. Only the top contributors to the separation with VIP score > 1 are shown, all of which are higher in cord blood. A fivefold cross validation (CV) of the model resulted in CV accuracy of 85%. This model outperformed 93% of label-shuffled random models (Wilcoxon p = 0.07). (**E**) The ML-PLSDA scores plot depicts differences in antibody profiles in maternal:cord PT pairs. In this orthogonalized model, the separation of PT maternal and cord antibody profiles was captured on LV1, capturing 73% of the Y variation. LV2 captures 0% of Y var variation. (**F**) VIP scores for loadings on LV1 (VIP scores > 1) highlight higher antigen-specific antibody levels in maternal plasma. A fivefold cross validation of the model resulted in a CV accuracy of 69%. The model outperformed 85% of models based on randomly label-shuffled inputs (Wilcoxon p = 0.15).

To further define the specific features that were most highly perturbed in PC1 (variation across time) and PC5 (variation by gestational age), loadings on each PC were plotted for each measured variable, where each dot represents an antigen-specificity grouped by each Fc-measurement. The greatest temporal changes were observed among IgG/IgG1 antibodies across nearly all antigen-specificities, representing the dominant transferred antibody population from maternal:infant (Fig. 3A). In addition, these IgG differences tracked with significant alterations in Fc-receptor binding profiles, that evolve uniquely over time. Less variation in FcRn, largely involved in early transfer, followed by FcγR (Fig. 3A) was observed over time, although some variation was notable across specificities.

Gestational age variation was examined using PC5 scores (Fig. 3B). Strikingly less variation was observed in this dimension, although variation was largely restricted again to IgG/IgG1 and Fc-receptor binding profiles. Interestingly, variation along PC5 was largely uniform for each Fc-receptor highlighting conserved principles of Fc-receptor mediated transfer across gestational ages. However, differences in transfer were noted for a specific subset of antigen-specificities. The top two antigen-specificities, which were consistently higher in PT (positive loading on PC5) across Fc measurements, included nBosd8 (Bovine milk allergen) and diphtheria.

To therefore define the particular specificities that were differentially transferred across gestational age groups, a multi-level discriminant analysis (ML-PLSDA)^21,24^ was used in mothers and their infants in each group across IgG levels (Fig. 3C–F). Robust separation was observed across FT mothers and infants in antibody profiles (Fig. 3C). The top antibody features that drove this separation were all enriched in cord blood and included higher levels of tetanus, norovirus, influenza, polio, respiratory syncytial virus (RSV), pertussis toxin, adenovirus, pneumo, cytomegalovirus (CMV), and auto-antigen (histone H3)-specific antibodies in the FT cords (Fig. 3D). Separation was also observed in maternal:cord antibody profiles in PT infants (Fig. 3E), however the antibody profiles that drove this separation were associated with higher levels of antibodies in the maternal blood (Fig. 3F). These data point to antigen-specific variation in transfer rates across the maternal:cord dyads. However, what governs these transfer differences selectively at different stages of gestation remain unclear but could point to opportunities to enhance transfer.

### Differences in PT and term antibody profiles diminish over time

**Figure 4:**
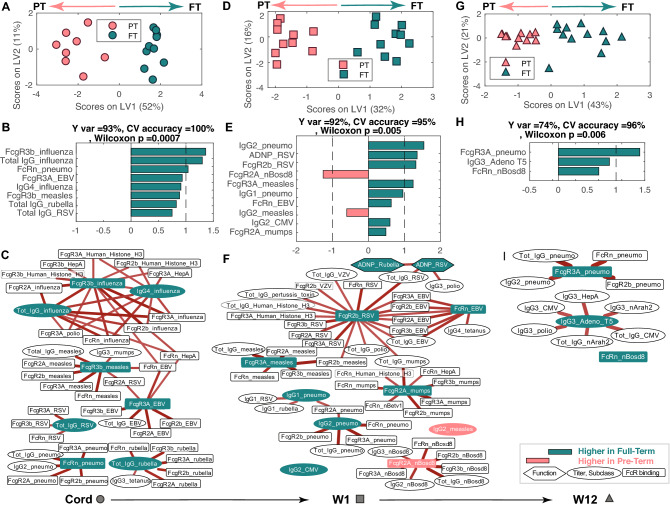
Antigen-specific antibody differences across gestational ages normalize over time but may point to immune vulnerabilities. (**A**) OPLSDA shows the separation between PT (PT:pink) and FT (FT:green) cord blood. Each dot represents an individual sample. The orthogonalized approach ensured that Latent Variable 1 (LV1) captured the separation between PT and FT antibody profiles while simultaneously capturing 93% of the Y-variation, while LV2 captured the antibody profile variances that do not contribute to this separation. fivefold cross validation was performed, resulting in 100% Cross Validation (CV) accuracy. Permutation test was performed by randomly shuffling the labels. Our model performed significantly better than random (Wilcoxon p = 0.0007). (**B**) The ordered VIP scores on LV1 for all the LASSO selected features are directed (and colored) based on whether the features are enriched in FT (green, right) or PT (pink, left) infants. (**C**) The network represents antibody features that are highly correlated with the LASSO-selected features across cord samples. (**D**, **E**) OPLSDA model shows the differences in antibody profiles between PT and FT infants at week 1, resulting in 95% CV accuracy and permutation test Wilcoxon p = 0.005. LV1 captured the separation of PT and FT, 92% of the Y-variation. Bar graph shows VIP scores for loadings on LV1 similar to (**B**). (**F**) The network represents features that are highly correlated with the LASSO-selected features across week 1 samples. (**G**, **H**) OPLSDA model separates PT and FT 12-week infants, resulting in 92% CV accuracy and permutation test Wilcoxon p = 0.006. LV1 captured 74% of Y-variation. The VIP scores plot highlights fewer differences between the groups. (**I**) The network represents antibody features that are highly correlated with the LASSO-selected features across 12-week infants. In networks of (**C**), (**F**), (**I**), edges depict correlation coefficients after removing correlation coefficients < 0.7 and p-values > 0.05 (corrected for multiple comparisons using Benjamini-Hochberg method). The red color of the edges represents positive correlation.

To begin to define the specific differences, and drivers of differences in antibodies transferred to FT and PT infants, we next compared the overall antibody profiles across the two groups of maternal:infant pairs over time (Fig. 4, Fig. S3). Classification models were generated per time point, initially using a least absolute shrinkage and selection operator (LASSO) to down-select features (to avoid overfitting) followed by Orthogonalized Partial Least Squares Discriminant Analysis (OPLSDA) using the LASSO-selected features. Robust separation was observed in infant profiles in the cord (Fig. 4A) that persisted but became less different over the first weeks (Fig. 4D) and months of life (Fig. 4G). The discriminating antibody profiles at birth were associated with higher levels of antibody features (Fc-receptor binding and levels) in the term infants (Fig. 4B). Given that the models select a minimal set of antibody features that are most distinct across the groups, we next examined the co-correlates of the model selected features to gain deeper insights into the antibody profiles differences across the infant groups. Strikingly nearly all the model selected features were tethered to additional FcR binding antibody features (Fig. 4C) pointing to the pivotal selective role and collaboration between FcRn and FcγRs in placental transfer at birth. Univariate analyses comparing PT and FT cord samples demonstrated similar differences between the two groups. Specifically higher measles, rubella, influenza, pneumo, RSV, and EBV-specific total IgG antibodies were observed in the cord of FT children (Figure S3).

Similarly, antibody profiles after 1 week of life also demonstrated robust separation (Fig. 4D), marked largely by enhanced antibody levels in the FT infants (Fig. 4E). However, a few features were enriched among PT infants (Fig. 4E). To gain further insights into the additional markers that tracked with the model-selected features, two large networks appeared around the features that marked the FT antibody profiles, composed of high IgG titers to nearly all tested antigens, as well as largely persistent FcγR binding antibodies to EBV, mumps, polio, RSV, VZV, and rubella. Moreover, a robust cluster of pneumo-specific and mumps-specific antibody features were also noted in the FT infants 1 week after birth, highlighting robust maintenance of these antibody-specificities to the newborn. Conversely, PT infants exhibited a highly selective enrichment of all antibody qualities to nBosd8, a milk-allergen (Fig. 4F).

Despite the normalization of antibody profiles with time across the babies (Figs. 1 and 2), antibody profiles still differed across FT and PT infants after 3 months of age (Fig. 4G), marked by a very small number of features in the FT neonates (Fig. 4H). The features pointed to a robust persistence of pneumo-specific antibodies of multiple qualities in FT infants as well as the presence of a highly persisting cluster of IgG3-antibodies to Adenovrius T5, CMV, polio, and peanuts (Fig. 4I). These data point to early differences in antibodies across PT and FT infants, that largely normalize, but that result in rare cases in persisting vulnerabilities in PT infants to bacterial pathogens known to cause enhanced disease in this vulnerable population. Thus overall, while higher levels of antibodies across specificities clearly distinguish term from PT infants, this difference is lost overtime, with few, but potentially important, differences persisting between the two groups months after birth.

### Evolution of transfer profiles during pregnancy

**Figure 5:**
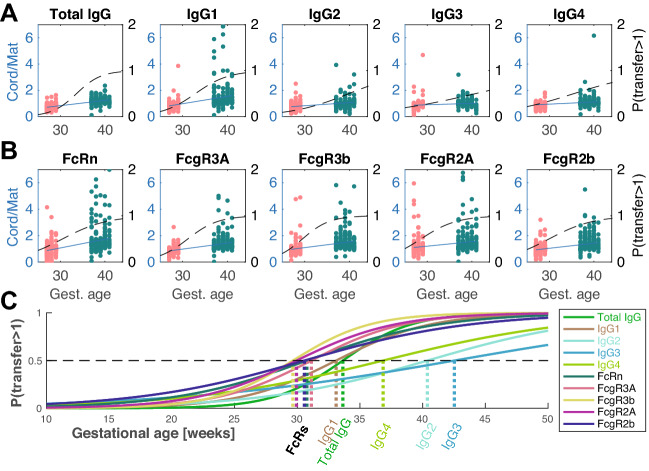
Preferential and early transfer of functional antibodies. (**A**, **B**) Transfer ratios (cord/maternal) per person-antigen are shown in pink and green for PT and FT newborns, respectively. Each dot represents the transfer ratio of an antigen-specific measurement for a maternal:cord pair plotted based on gestational age. The ratios where maternal levels were below PBS levels as well as measurements with transfer ratios > 7 were excluded from the analysis for more stable results. Linear regression lines are overlaid in blue. The regression coefficients for total IgG, IgG1, IgG2, IgG3, IgG4, FcRn, FcR3A, FcR3B, FcR2A and FcR2B were calculated to equal 0.04, 0.07, 0.06, 0.02, 0.06, 0.09, 0.05, 0.05, 0.04, and 0.03, respectively. Additionally, logistic regression was performed and the probability of transfer > 1 was calculated and plotted in dashed black curves (right y axis). (**C**) The individual probabilities for each Fc-readout was plotted separately and overlaid for comparison. The likelihood of transfer ratio ≥ 1 surpasses the likelihood of transfer ratio < 1 was marked on the graph with hatched lines for each Fc-readout.

To ultimately define whether antibody transfer occurs via the same mechanism(s) in PT and FT infants, we next plotted transfer ratios for all specificities for each analyzed feature according to gestational age (Fig. 5). A temporal transfer curve (blue solid line) was then calculated for each feature using linear regression. Specifically, a nominal multinomial logistic regression was used to calculate the probability that cord values would exceed maternal levels, where dashed black lines represent the overall temporal shift (left y axis, Fig. 5A,B). As expected, total IgG transfer ratios increased with gestational age (Fig. 5A), largely for IgG1 antibodies. More moderate increases were noted in the transferred slopes of other IgG subclasses IgG2-4, consistent with a total increase overtime but no evidence of preferentially enhanced transfer, as is observed for IgG1 antibodies.

To begin to define the mechanism underlying transfer differences over time, we next examined the transfer profiles for Fc-receptor binding antibodies. A steep and linear increase in FcRn binding antibodies was observed over time (Fig. 5B) as expected with increases in transferred antibodies with gestational age. Similarly, a steady increase in FcγR2B binding antibodies was observed. Conversely, a more striking logistic growth was observed for FcγR3A,B receptor-binding antibodies (Fig. 5B).

To gain a clearer sense of the timing of antibody selectivity across gestational ages, the gestational age when the likelihood of the transfer ratios increased above 1 (≥ 1) was calculated using a logistic regression (black dashed lines in Fig. 5A,B, replotted and overlaid in Fig. 5C). For each feature, the gestational age when this likelihood exceeded 0.5 was calculated (Fig. 5C, shown on horizontal axis). Surprisingly, all Fc-receptor binding properties cross the transfer threshold earliest in pregnancy, highlighting the critical selective influence of Fc-receptors in shaping transfer. FcγR3B, followed by FcγR2A, FcRn crossed the transfer threshold earlier than IgG1 levels. These curves were followed closely by the total IgG and other IgG subclasses, highlighting placental kinetic transfer differences across IgG-subpopulations and IgG subclasses. Despite the higher affinity of IgG3 for many Fcγ-receptors, the preferential transfer of IgG1 throughout gestation argue for a collaboration between FcRn and Fcγ-receptors expressed within the placenta. Collectively the data therefore argue for early collaboration between FcRn and FcγRs in driving preferential transfer of highly functional antibodies early in gestation, followed by enhanced overall antibody transfer over time.

### Sequential preferential transfer of functional antibodies over gestation

**Figure 6:**
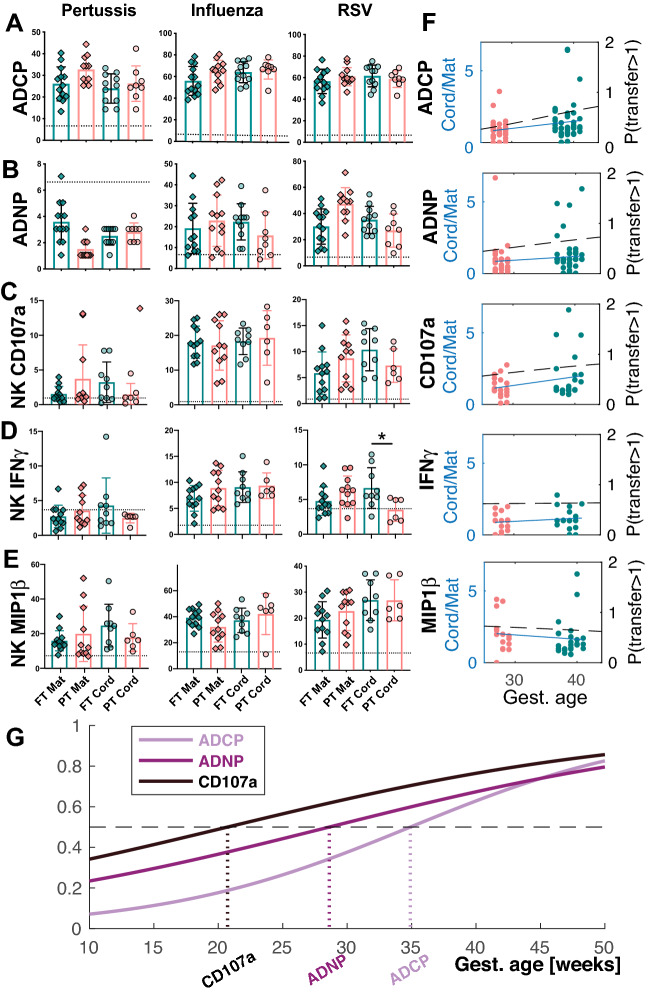
Functional antibodies transfer early in pregnancy. (**A–E**) The dot plots depict the univariate level of (**A**) ADCP, (**B**) ADNP, (**C**) antibody mediated NK cell degranulation (NK CD107a), (**D**) antibody driven NK cell IFNγ secretion, (**E**) antibody dependent NK cell chemokine MIP1β secretion across Pertussis-, Influenza-, and RSV-specific antigens. (**F**) The dot plots show the transfer ratios by gestational age, with each dot representing a different antigen and sample set organized by gestational groups (PT: pink and FT: green). Fewer antigens were included for the functional profiling due to sample limitations. Regression coefficients were estimated as 0.08, 0.10, 0.01, − 1.41, and 0.08 for ADCP, ADNP, NK MIP1β, IFNγ, and CD107a expression, respectively. Moreover, the likelihood of transfer ratios surpassing the likelihood of transfer ratio < 1 were calculated at gestational week 36, 41, 63, 36 and 24, respectively. (**G**) The individual probabilities for ADCP, ADNP, and NK CD107a were plotted and overlaid for comparison. The probability curves for NK IFNγ and MIP1β secretion were excluded since the fitted nominal multinomial logistic regression model was not reliably parameterized. The likelihood of transfer ratio ≥ 1 surpasses the likelihood of transfer ratio < 1 was marked on the graph with hatched lines.

The early Fc-receptor transfer profiles suggested that the placenta may select for highly functional antibodies early during gestation. To test this hypothesis, we next examined the overall levels of functional antibodies across the maternal:infant pairs (Fig. 6). The levels of ADCP, ADNP, and antibody dependent NK cell activating (NK cell degranulation and cytokine secretion, ADNK) inducing antibodies were screened across pertussis, influenza and RSV. Surprisingly, despite differences in the overall levels of IgG-specific antibodies to each of these targets across the PT and FT cord samples (Fig. 5A), antibody effector function was largely concordant across most FT and PT infants, with the exception of RSV-specific NK IFNγ secretion (Fig. 6A–E). For example, nearly equivalent antibody-dependent cellular phagocytosis, driven largely by FcγR2^25–28^, was observed across PT and FT cords (Fig. 6A). Similarly, equivalent levels of neutrophil-phagocytosis activating antibodies were observed across PT and FT infants to all tested pathogens (Fig. 6B). Conversely, NK cell activating antibodies increased more variably over time, with early robust transfer of Influenza-specific antibodies able to drive NK cell activation in PT infants (Fig. 6C–E). Thus, despite lower level IgG-levels early in gestation, these data suggest early phagocytic antibody transfer during gestation.

Accordingly, to define whether particular functions were transferred preferentially, transfer slope analysis was performed (Fig. 6F). For phagocytic functions (ADCP and ADNP), slopes were relatively stable (Fig. 6F), consistent with the early transfer of FcγR2 (Fig. 5B,C), suggesting that transfer increased overtime, aimed at populating the infant early on with functional antibodies. Conversely, the regressed ADNK curves were nearly flat and even decreased for antibody mediated NK cell chemokine secretion (MIP1β) with gestational age, suggesting a potential perpetual selection, from very early gestation, aimed at populating the infant with these highly functional antibodies. Moreover, logistic regression of the probability of transfer > 1 (black dashed lines in Fig. 6F) suggested that antibody-mediated NK activating antibodies are likely to be preferentially transferred as early as week 24 of gestation (Fig. 6G). These data suggest that functional antibodies are transferred early and consistently throughout pregnancy, to ensure in utero protection and increased probability of survival. Moreover, these data also point to a potentially preferential transfer of NK cell activating antibodies earliest in gestation, likely linked to the enhanced maturity of neonatal NK cells in early life ^29^.

### Selective antigen-specific antibody decay

**Figure 7:**
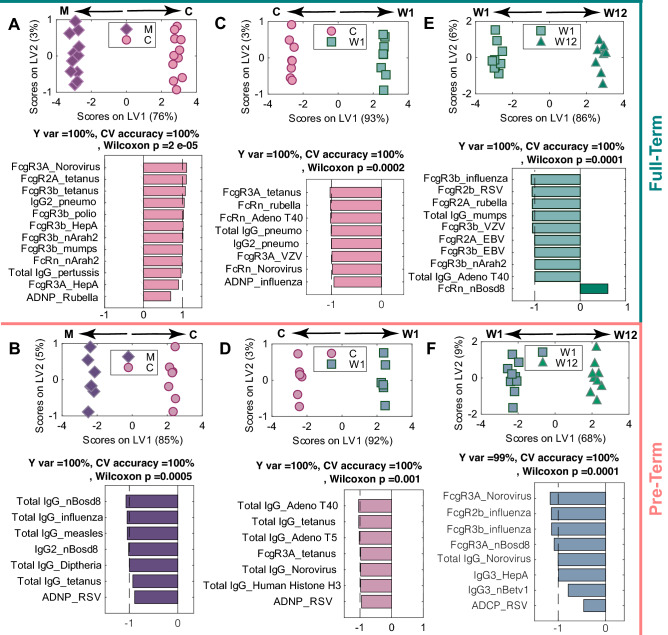
Similar antigen-specific decay patterns occur following birth in PT and FT infants. Separate ML-PLSDA models were constructed for PT and FT infants using LASSO-selected features to examine the evolution of antibody profile changes over time in the maternal:cord (**A**, **B**), across cord:week 1 (**C**, **D**), and between week 1 and week 12 (**E**, **F**). (**A**) The ML-PLSDA scores plot model separating maternal and cord antibody profiles in FT neonates is shown on top. The model captures 100% of the Y-variation. The bar plot depicts the VIP scores for loadings on LV1, which are signed and colored to reflect which group they are enriched in. The dashed line marks the threshod of VIP score = 1 above which are the top contributors to the separation. A fivefold cross validation of (CV) the model resulted in CV accuracy of 100%. This model significantly outperformed label-shuffled random models (Wilcoxon p = 2 × 10^–5^). (**B**) The ML-PLSDA scores plot shows the separation in maternal and cord antibody profiles in PT children. The bottom bar plot illustrates the VIP scores for loadings on LV1. The bars point to the direction of higher abundance, which are dominantly found in the maternal plasma. A fivefold cross validation of the model resulted in a CV accuracy of 100%. The model outperformed models based on randomly label-shuffled inputs (Wilcoxon p = 0.0005). (**C**, **D**) Similar models are shown for cord and week 1 profiles for full-term and PT infants are depicted in Panels with a CV accuracy of 100% for both models, with permutation test Wilcoxon p-values of 0.0002 and 0.001 for PT and FT infants, respectively. (**E–F**) The ML-PLSDA models show the week 1:week 12 profiles for FT and PT children with CV accuracy of 100% and Wilcoxon p-values for the permutation testing of 0.0001 for both models. IgM, IgA1, and IgA2 are excluded from all analyses to focus on IgG-specific trends.

Finally, we aimed to define the decay patterns of antigen-specific antibodies overtime following birth across both PT and FT infants by comparing antibody profiles across each set of timepoints (Fig. 7). As expected in the FT infants, at birth, the presence of higher levels of particular antibody populations were enriched in the cord, due to preferential transfer across the placenta, marked by enhanced levels of Fc-receptor binding antibodies to norovirus, tetanus, S. pneumoniae, poliovirus, hepatitis A, mumps, and allergens, total pertussis antibody titer as well as ADNP-capable antibodies to rubella (Fig. 7A). As expected, with time, antibody levels decayed, highlighting enriched antibody levels at early timepoints (C > W1 and W1 > W12). However, the decay occurred disproportionately across antigen-specificities (Fig. 7C,D). Interestingly, ADNP inducing antibodies to influenza, FcγR3A-NK cell activating antibodies to tetanus and Varicella zoster virus, FcRn binding antibodies to adenovirus T40 and norovirus, and overall levels of S. pneumoniae antibodies were preferentially lost over the first week of life (Fig. 7C). Further loss of total IgGs to mumps, adenovirus T40, and Fc-receptor binding antibodies to influenza, RSV, Varicella zoster virus, EBV, and peanut allergen were observed later overtime, whereas FcRn binding bovine milk allergen slightly increased by the third month (Fig. 7E). These data suggest an early preferential loss of the most functional antibodies and a potentially slower decay in overall levels of IgG in FT infants.

Unlike FT comparisons, higher levels of antibodies were observed in the maternal circulation compared to cord (Fig. 7B), marked by higher levels of cow milk-, influenza-, measles-, tetanus- and diphtheria-specific antibodies and ADNP-inducing antibodies to RSV in the maternal blood (Fig. 7B). An early loss of ADNP antibodies was observed to RSV. Loss of FcγR3A-NK cell activating tetanus antibodies occurred soon after birth along with an early loss of total tetanus-, influenza-, adenovirus-, norovirus-, and human histone H3-specific antibody decay (Fig. 7D). In premature infants, the next 3 months was associated with a decay in FcγR binding antibodies to influenza, Norovirus, and cow milk (Fig. 7F). Additionally, total IgGs declined to norovirus over this second phase, and a loss of ADCP was also observed for RSV. Interestingly, IgG3 antibodies to HepA and pollen were lost in this later phase following birth. These data highlight a selective loss of some antibody subpopulations early (ADNP and tetanus/adeno) and a later decline in Fc-receptor binding, functional, IgG3 antibodies following several months of birth, highlighting different potential windows of vulnerability following birth across FT and PT infants.

Thus, collectively, our findings confirm the presence of higher transferred antibodies in FT infants but point to placental mechanism(s) that aim to transfer functional antibodies early during pregnancy to empower the neonate to fight infection even if born prematurely. The data also point to differential kinetic vulnerabilities in PT and FT infants that have critical implications for the design of next generation of vaccines or therapeutics able to leverage the unique filtering features of the placenta that clearly evolves over the course of pregnancy to empower neonates with the greatest chance of survival after birth.

## Discussion

In 2018, 5.3 million deaths occurred in children under 5 years of age, with 47% of these reported in the first month of life^30^. The leading cause of death in these children was attributed to complications of PT birth, making the early months of life an especially vulnerable period for those born early. PT infants are particularly susceptible to infection, especially vulnerable to pathogens affecting the respiratory tract^2^. While perturbed mucosal and cellular immunity, which mature with age^7^, are likely key to this vulnerability, PT infants receive fewer placentally transferred antibodies, that have also been implicated in compromised immunity. However, recent data suggest that despite these lower titers, PT newborns possess similar anti-viral antibody repertoires and equivalent levels of neutralizing antibodies, suggesting that despite reduced placental transfer, placental mechanisms must exist to ensure robust transfer of broadly reactive and functionally relevant antibodies^16^. Using a systems serology approach, we observed significant differences in the overall magnitude, but not the functional or Fc-receptor binding quality of antibodies across PT and FT neonates, arguing for conserved and very early Fc-receptor mediated selection of the most functional antibodies for transfer to neonates.

Mechanistically, the change in sieving may occur via differential expression of Fc-receptors or via the changes in the localization of Fc-receptor expression. Importantly, multiple Fc-receptors have been observed in the placenta^31–34^, and recent data have noted co-localization of FcγR3A with FcRn on synciotrophoblasts that may explain the preferential transfer of NK-cell activating antibodies^21^. However, here the data point to earlier and steady transfer of both NK cell activating and phagocytic antibodies over time, pointing to the selection of sub-populations of IgG1s from maternal circulation to populate the neonatal system. Given IgG1 antibodies are preferentially transferred compared to additional IgG subclasses, *e.g.* IgG3, that bind to Fcγ-receptors with higher affinity, these data suggest that FcRn must be involved in the selection. As FcRn typically functions as a homodimer^35–38^, requiring two receptors to trap an antibody, it is also plausible that FcRn may form a heterodimer with additional Fcγ-receptors within synciotrophoblasts, aimed at capturing the most functional IgG1-antibodies from maternal blood. Conversely, as gestation progresses, the collaboration between FcRn and other Fc-receptors may change. Evolutionarily, this transient selective transfer process might occur to equip infants with the highest quality antibodies able to bind to FcRs on immune cells to elicit functional responses at birth, poised to fight infection even if born prior to full gestation.

The placenta is a complex organ consisting of chorionic villi, which are surrounded by chorion. Antibodies in the maternal blood flowing through the intervillous space are first transported through the outer syncytiotrophoblast layer of the chorion to the inner layer of cytotrophoblast progenitor cells and the fetal endothelium into the fetal capillaries. However, the fetal endothelium also expresses FCγR2B, which is thought to play an additional role in sieving antibodies prior to transfer into cord blood^31,39^. However, additional Fc-receptors are expressed dynamically in the placenta in the setting of infection^40^, likely all contributing to changes in the quality of transferred antibodies. Specifically, dynamic cellular heterogeneity of the placenta was previously shown^40^, that collectively could shift the quality of antibodies that are allowed to transit across the maternal/fetal barrier. Along these lines, several infections have been linked to compromised placental transfer^19,41^, including HIV^42–44^, malaria^43,45–48^, and Zika^49^ likely occurring both due to altered humoral immunity in the infected mother, but also due to changes in the placenta that may shift the quality of the captured antibodies. Of all the antibody specificities, only cow-milk allergen specific antibodies were significantly enriched in PT neonates one week after birth compared to FT infants. Exposure to allergens have been controversially linked to allergies in PT infants^50–53^. However, emerging data point to antibodies as a robust surrogate of food allergy. Thus, future studies, aimed at profiling antibody transfer will be able to identify the relationship of food-antibodies and allergy across gestational ages.

Interestingly, previous studies highlighted the transfer of equivalent antibody repertoires and neutralization in PT and FT infants^16^, largely controlled at the antigen-binding domain level of the antibody (the Fab). However, how Fab selection is regulated via the placenta is unclear. Emerging data point to the co-evolution of the Fab and Fc-domain, in such a way that the Fab and Fc domain often collaborate to mediate protective immunity^54–56^. For example, in the context of Influenza infection, several of the most potent protective antibodies require Fc-mediated activity to mediate protection from infection in animal models^57–59^. Similarly, many protective HIV-neutralizing antibodies^60–63^ and bacterial toxin-specific antibodies^64,65^ also require Fc-mediated activity in order to mediate protection in animal models. Thus, it is plausible that the placenta aims to leverage the most highly protective antibodies that both neutralize and drive Fc-effector function to maximize protective immunity in the neonate.

One limitation of the current study is the small homogeneous sample size, which was alleviated by selecting a cohort of swedish pregnant women that experienced extremely PT births to increase the effect size, improve the power of our study, and to reduce geographic and environmental confounders. While this selective population level enrichment helped highlight differences between PT and FT dyads, the results only provide general clues related to the timing where transfer of functional antibodies changes throughout gestation. As such, future studies with higher numbers of dyads spanning the full range of extremely PT to FT pregnancies will provide an opportunity to dissect the precise gestational timing of placental transfer changes. However, whether the levels of functional antibodies transferred to PT infants are sufficient to prevent disease remains unclear, as PT infants remain vulnerable to several mucosal infections^2,9^, but this selection of functionally enhanced antibodies against pathogens may provide some evolutionary survival advantage aimed at giving the neonate the high probability of survival after birth.

In this study, maternal and infant immunization status was kept similar between the PT and FT dyads. However, infant vaccination in early life may alter antibody profiles and help normalize profiles across the PT and FT infants. In fact, a similar boost was observed across tetanus-, diphtheria-, and pertussis toxin-specific antibodies in both PT and FT children at three months of age, highlighting the power of immunization in early life in boosting immunity to lethal pathogens. Moreover, understanding the mechanism(s) of transfer thoughout gestation compounded with our growing appreciation for the evolutionary changes and maturation of the cellular and organ development that occurs in the first few days to years of life, may lead to novel strategies to maximally harness immunity in early life to fight infections and disease. Thus, while the scope of the current study was focused on solely exploring changes in the plasma antibodies, future studies aimed at examining the intersection of antibody, innate immune cellular, and adaptive immune evolution may shed more light on the design of next generation vaccines and/or therapeutics able to leverage the unique evolving biology of the placenta to maximally protect infants as they enter the world.

## Methods

### Antigen-specific antibody isotype, subclass, and FcR binding

All maternal and cord samples were collected as pure plasma. The plasma was collected by centrifugation prior to the isolation of PBMCs, that were purified by Ficoll-Hypaque separation (for other studies such as^8^). All samples were collected in sodium-heparin tubes (BD Vacutainer CPT vial). Antigen-specific isotypes, IgG subclasses, and binding to FcγRIIA, IIb, IIIA, IIIb and FcRn were measured as previously described^66,67^. Protein antigens (Table S1) were covalently coupled to MagPlex© microspheres via a two-step carbodiimide reaction. The beads were activated with 80 µL of activation buffer (0.1 M NaH_2_PO_4_, pH 6.2), 10 µL of 50 mg/mL Sulfo-NHS (N-hydroxysulfosuccinimide, Pierce, A39269), 10 µL of 50 mg/mL ethyl dimethylaminopropyl carbodiimide hydrochloride (EDC) and incubated for 30 min at room temperature. The beads were washed three times in coupling buffer (0.05 M morpholinoethanesulfonic acid (MES), pH 5.0), then incubated with protein antigen in coupling buffer for two hours at room temperature. The beads were subsequently washed and blocked with PBS-TBN (PBS, 0.1% BSA, pH 7.4), then washed with PBS-Tween Buffer (PBS, 0.1% BSA, 0.02% Tween 20, 0.05% Azide, pH 7.4). Beads were resuspended in PBS at concentration of 5 × 10^6^ beads/mL and stored for up to two weeks at 4° C.

To measure isotypes and subclasses, samples were diluted 1:10 in PBS to achieve a final assay dilution of 1:100 when 5 µL sample was added to 45 µL antigen-coated beads at a concentration of 5 × 10^6^ beads/mL. Immune complexes were incubated for 16 h overnight shaking at 900 rpm at 4 °C. Unbound antibody was removed via washing six times in Luminex assay buffer with an automated plate washer. PE-conjugated secondary antibodies (IgG, IgG1, 2, 3, 4, IgA1, 2, IgM) were diluted 1:500 in Luminex Assay Buffer, and 40 µL were added and incubated for 1 h shaking at 900 rpm at room temperature. Unbound secondary was washed, and Qsol Buffer was added for reading on the Intellicyt iQue. Relative levels of isotypes and subclasses were calculated as the median fluorescence intensity of PE. Background was determined by a no-antibody control.

To prepare Fc receptors, AVI-Tagged FcγRs and FcR were biotinylated with BirA enzyme (Avidity, BirA500) for 30 min rotating at room temperature, and excess biotin was removed with Zeba Spin Desalting Columns, 7 MKCO (Thermo Fisher, 89892). Immediately prior to use, biotinylated FcRs were coupled to Streptavidin-PE for 10 min. Immune complex binding to FcRs was then measured and calculated as described for isotypes and subclasses.

### Antibody-dependent cellular phagocytosis (ADCP)

Based on the published protocol^68^, Rubella, pertussis, influenza, pneumococcus, CMV, and RSV antigens were biotinylated with LC–LC biotin and coupled to yellow-green fluorescent Neutravidin 1 µm beads (Thermo Fisher, F8776) for 2 h at 37 °C. Coupled beads were then washed twice with 5% BSA/PBS and stored at 4 °C for up to 1 week. Cord and maternal samples were diluted 1:100 and 10 µL sample was incubated with 10 µL antigen-coupled beads for 2 h at 37 °C to form immune complexes. After washing with PBS to remove unbound non-specific antibody, THP-1 monocytes were added (200 µL/well) at a concentration of 1.25 × 10^5^ cells/mL and incubated with immune complexes overnight for 16 h at 37 °C. Cells were fixed with 4% PFA and acquired with an Intellicyt iQue. Phagocytosis was measured by a phagocytosis score ((gMFI of bead-positive cells × percentage of bead-positive cells)/1000). Background levels of phagocytosis were measured with a no-antibody control for each antigen.

### Antibody-dependent neutrophil phagocytosis (ADNP)

Based on the published protocol^69^, Rubella, pertussis, influenza, pneumococcus, CMV, and RSV antigens were coupled to beads, and antigen-coated beads were incubated with sample as described for ADCP. The neutrophils used in these assays were from healthy adult donors (not the infants). To prepare PBMCs from whole blood, erythrocytes from were lysed (Buffer, 150 mM NH_4_Cl, 10 mM KHCO_3_, mM Na_2_EDTA in 1 mL H_2_O), and leukocytes were washed twice with ice cold PBS. PBMCs were added (200 µL/well) at a concentration of 2.5 × 10^5^ cells/mL. Neutrophils were stained with a Pacific Blue conjugated anti-CD66b secondary antibody (BioLegend, 305112). Finally, cells were fixed and acquired, and a phagocytosis score was calculated as described for ADCP.

### Antibody-dependent natural killer (NK) cell degranulation assay

ELISA plates were coated with 100ul/well of pertussis, influenza A, and RSV antigen at 2 µg/mL each and incubated 2 h at 37 °C. Plates were washed three times with PBS and blocked with 5% BSA/PBS overnight at 4 °C. NK cells were isolated from Buffy coats from 3 healthy adult donors (not cord or infant blood from the study) using RosetteSep NK cell enrichment kit (StemCell Technologies) and rested overnight in the presence of IL-15. The following day, samples were diluted 1:25 and 50 µL/well diluted sample was added to ELISA plates after washing, incubating at 37 °C for 2 h. A CD107 PE-Cy5/BFA/GolgiStop (BD Biosciences, 555802) cocktail was prepared and added directly to NK cells before adding 200 µL/well NK cells at a concentration of 2.5 × 10^5^ cells/mL. Cells and plate-bound immune complexes were incubated for 5 h at 37 °C. After incubation and washing, NK cells were stained for surface markers, CD56 PE-Cy7, CD16 APC-Cy7, and CD3 Pacific Blue (BD Biosciences, 557747, 557758, 558124) in V-bottom plates. Cells were subsequently washed and incubated with PermA for 15 min followed by intracellular staining with fluorescently conjugated MIP1β PE and IFNγ FITC (BD Biosciences, 550078, 340449). NK cell activation and degranulation markers were measured by flow cytometry with the Intellicyt iQue.

### Principal component analysis (PCA)

A PCA model was constructed using 323 antibody features. Variables were centered and scaled to a standard deviation of 1. In the two-dimensional space of PC1 vs. PC5, temporal trends of samples were observed to be in the direction of PC1, whereas PC5 was observed to partially separate PT and FT profiles.

### Identification of PT/FT-specific signatures with LASSO and OPLSDA

The minimum signatures of antigen-specific antibody biophysical and functional measurements that could differentiate between PT and term infants at birth, week 1 and week 12 were identified using the Least Absolute Shrinkage and Selection Operator (LASSO) method^70^. This was implemented using Matlab software (version 2018a, Mathworks, Natick, MA) on the data, where each variable was centered and scaled to have a standard deviation of 1. Orthogonalized Partial Least Squares Discriminant Analysis (OPLSDA)^71,72^ was then used to visualize and assess the predictive ability of LASSO-selected biomarkers for classifying PT and FT groups. PLSDA is a multivariate regression technique where linear combinations of features are used to predict the variance in the dependent categorical variables. The model was then orthogonalized such that Latent Variable 1 (LV1) captured the variance in features that are in the direction of the pairwise separation of the groups, while other latent variables described the variation orthogonal to this predictive component. The LASSO + PLSDA feature reduction and discriminate analysis in a cross validated framework was previously described^60,73^. fivefold cross validation was performed on the data (100 random fivefold cross validation). To assess model significance, permutation test was performed on the cross validated models by randomly shuffling the labels. Networks of features correlated to the LASSO-selected features were then constructed (co-correlate networks). Combinations of these correlates can be considered as possible substitutes for the LASSO-selected features as biomarkers of group separations.

### Multi-level partial least squares discriminant analysis (MLPLSDA)

To mathematically identify the key features contributing to the profile differences between maternal and cord blood, MLPLSDA^24^ model was constructed. MLPLSDA uses the same principles as OPLSDA analysis for multivariate data but also takes advantage of the paired structure of the data (paired maternal and cord blood). Intuitively, this analysis subtracts the effect of inter-pair variability, i.e. the heterogeneity between maternal:cord samples and focuses on the effects within maternal:cord samples. The model was constructed using LASSO-selected variables. Variables were centered and scaled to a standard deviation of 1. fivefold cross validation was performed on the data (Venetian blinds). To assess model significance, a permutation test was performed by randomly shuffling labels.

### Construction of the correlation network of the LASSO-selected features

Spearman correlation of only the LASSO-selected Fc features to all original 323 antigen-specific antibody features were calculated. Each node is a feature and the thickness of the edges between nodes were weighted using significant correlation coefficients. The p-value depicting the significance of these correlations were corrected for multiple comparisons (Benjamini–Hochberg q-value < 0.05, testing the hypothesis of zero correlation). Only correlations with corrected p-values < 0.05 and Spearman correlation coefficients > 0.7 were included. Also, only the features directly and significantly correlated with the LASSO-selected features were included in the figure.

### Construction of the correlation heatmap

Spearman correlations of maternal and cord antibody measurements were calculated for PT and FT maternal-cord dyad separately. Correlation coefficients for IgG subclasses and binding to Fc receptors across 24 antigens were then depicted in a heatmap (Fig. S1). Due to the qualitative nature of this heatmap, statistical significance was then calculated without correcting for multiple comparisons.

## Supplementary Information

Supplementary Figure S1.

Supplementary Figure S2.

Supplementary Figure S3.

Supplementary Table S1.

Supplementary Information 5.

## Data Availability

All data generated or analysed during this study are included in this published article and its supplementary information (see Supplemental Data table).
