# Supplementary material for: Selective transfer of maternal antibodies in preterm and fullterm children
Source: Sci Rep. 2022 Sep 2;12:14937. doi: 10.1038/s41598-022-18973-4 (PMC9440225; doi:10.1038/s41598-022-18973-4)
Supplement: Supplementary file 2 — Supplementary Figure S2. [file 41598_2022_18973_MOESM2_ESM.pdf]

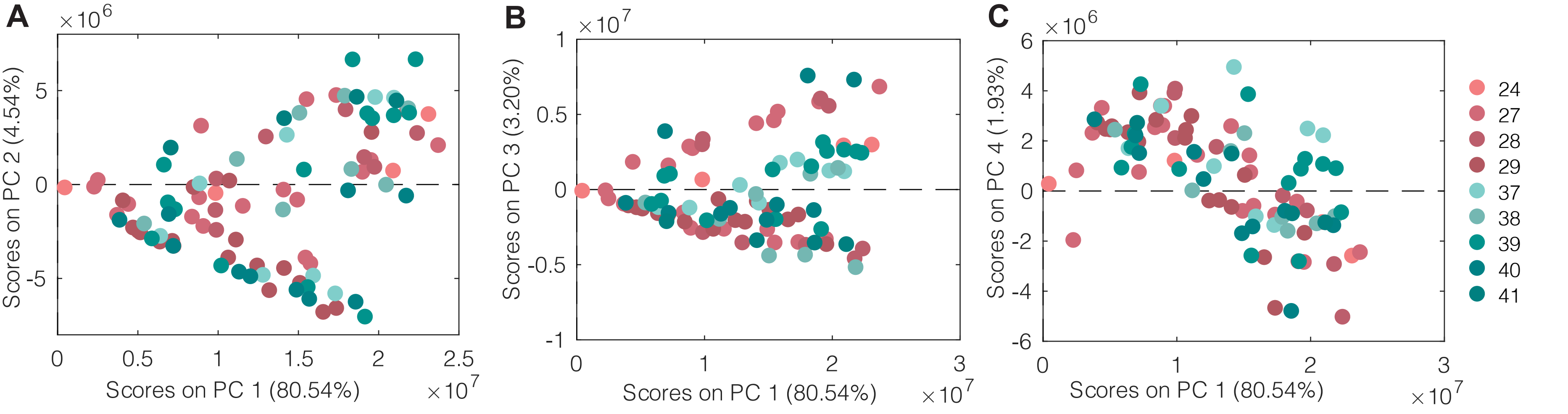

Figure S2. PC2-PC4 Do not capture the variability in data along the preterm-Fullterm axis. Scores plots for the PCA of Fig 2A-B were plotted for the PC1 vs. PC2 (A), PC2 (B), and PC3 to highlight the fact that the variability in the data across PC2-4 does not track with gestational age. The dot color depicts the gestational group of the sample.
