# Supplementary figures and images for: Selective transfer of maternal antibodies in preterm and fullterm children
Source: Sci Rep. 2022 Sep 2;12:14937. doi: 10.1038/s41598-022-18973-4 (PMC9440225; doi:10.1038/s41598-022-18973-4)

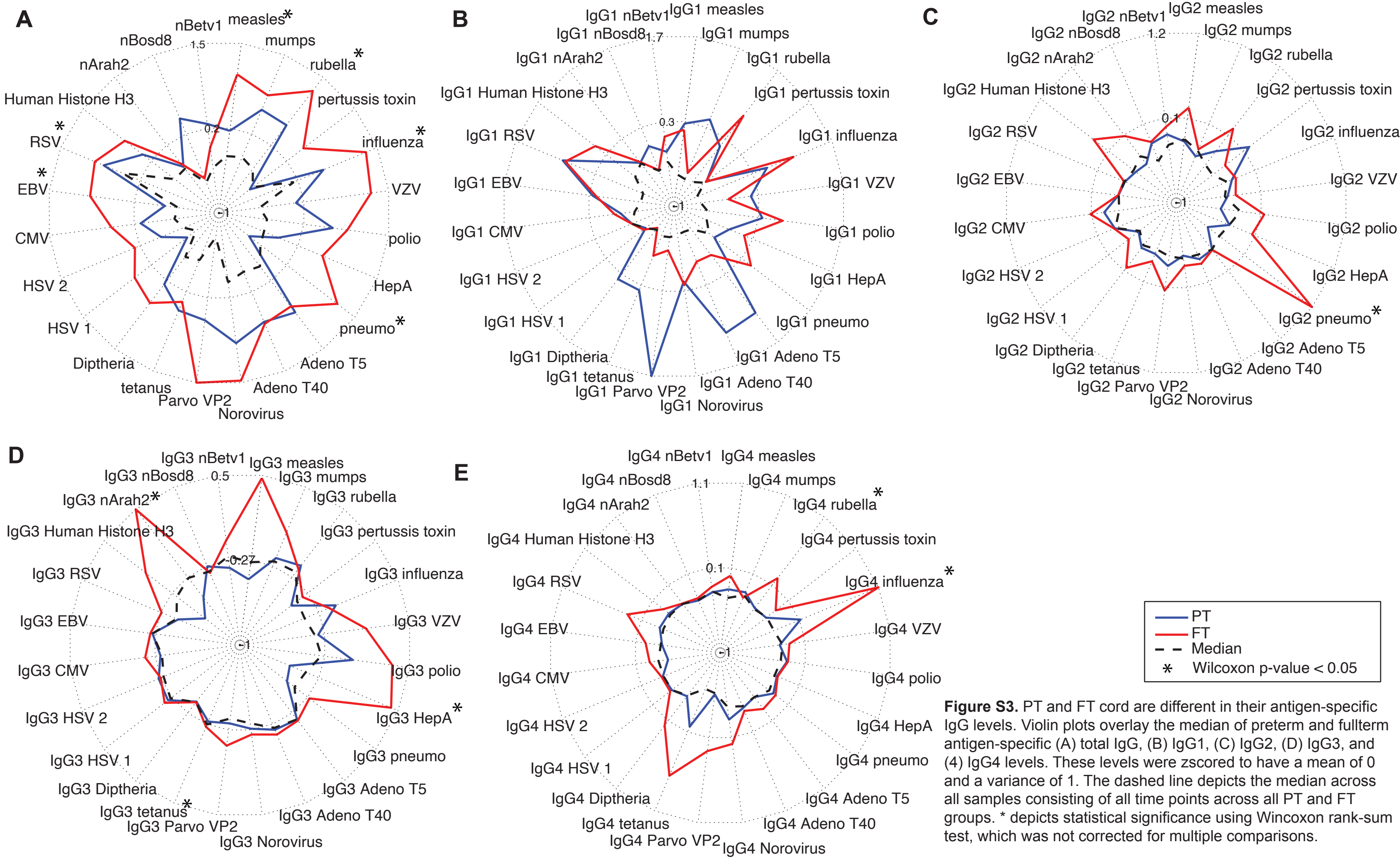

Supplement: Supplementary file 3 — Supplementary Figure S3. [file 41598_2022_18973_MOESM3_ESM.pdf]
