# Supplementary material for: Selective transfer of maternal antibodies in preterm and fullterm children
Source: Sci Rep. 2022 Sep 2;12:14937. doi: 10.1038/s41598-022-18973-4 (PMC9440225; doi:10.1038/s41598-022-18973-4)
Supplement: Supplementary file 4 — Supplementary Table S1. [file 41598_2022_18973_MOESM4_ESM.pdf]

Table S1. Antigen Reagents

Pathogen	Antigen	Vendor, Catalog #
Measles, Edmonton	Inactivated Virus	BioRad, PIP013
Mumps, Enders	Inactivated Virus	BioRad, PIP014
Rubella, HPV-77	Inactivated Virus	BioRad, PIP044
Varicella zoster virus (VZV)	gE(Orf68)	Made in house
Epstein barr virus (EBV)	gp350/220	Immune Technology, IT-005-035p
Herpes simplex virus (HSV)	gD (1), gC (2)	Immune Technology, IT-005-055p, IT-005-011p
Influenza A	HA H1N1 and H3N2	Immune Technology, IT-003-0042ΔTMp, IT-003-001p, IT-003-0011ΔTMp
Respiratory Syncytial virus (RSV)	Pre and post A and B	Barney Graham, NIH
Cytomegalovirus (CMV)	gH pentamer complex	CMV-PENT-100
Poliovirus	Polio vaccine salk inactive	MGH Pharmacy, 976210
Tetanus	Toxoid	UMass Medical Center
Pertussis, Bordetella	Toxin	List Biological Laboratories, #180
Hepatitis A	Vaccine, HAVRIX	MGH Pharmacy, 5SR75
Haemophilus influenzae B	Vaccine, Polysaccharide conj-tet	MGH Pharmacy, 2000696
Hepatitis B virus (HBV)	HBsAg adw	Genway, GWB-B11E0A
Pneumococcus	Pneumovax vaccine	MGH Pharmacy, 8524700
Adenovirus	Type 5 and 40	BioRad, MPP002; Native Antigen Co., A40P-100
Norovirus	Virus-like particle 1	Native Antigen Co., REC31620-100
Parvovirus	Virus-like particle 2	Native Antigen Co., PV-VP2-100
Diphtheria	Toxin	Native Antigen Co., DIP-TNL
Human Histone H3	Citrulline peptide	Abcam, ab32876
Peanut allergen	nAra h 2	Indoor Biotechnology, NA-AH2-1
Bovine milk allergen	nBos d 8	Indoor Biotechnology, NA-BD8-1
Birch pollen allergen	nBet v 1	Indoor Biotechnology, NA-BV1-1
