# Supplementary material for: Selective transfer of maternal antibodies in preterm and fullterm children
Source: Sci Rep. 2022 Sep 2;12:14937. doi: 10.1038/s41598-022-18973-4 (PMC9440225; doi:10.1038/s41598-022-18973-4)
Supplement: Supplementary file 1 — Supplementary Figure S1. [file 41598_2022_18973_MOESM1_ESM.pdf]

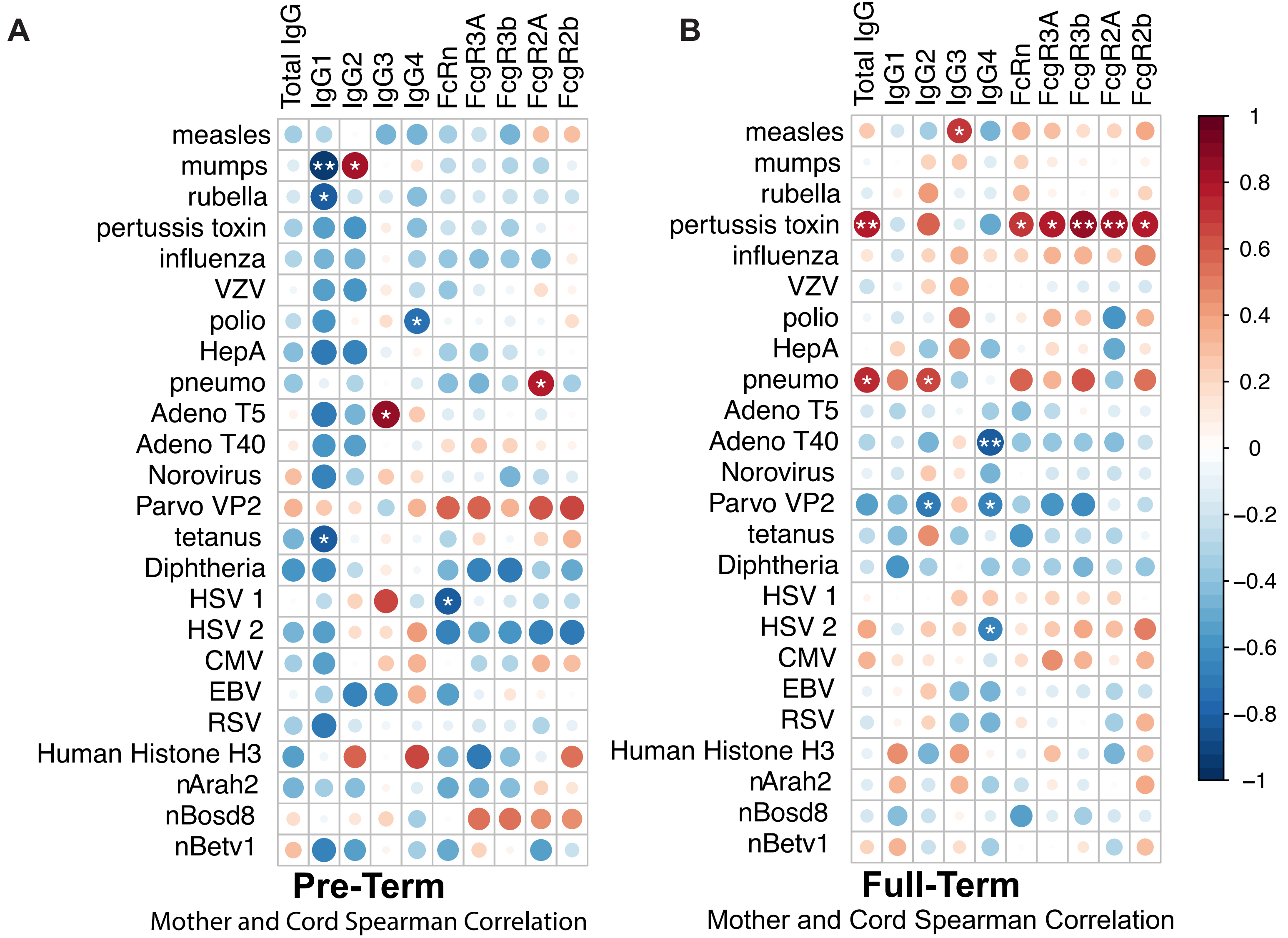

Figure S1. Differential coordination of antibody transfer to pre- and full-term infants. Spearman correlations of maternal and cord antibody measurements were calculated for (A) preterm and (B) fullterm maternal-cord pairs separately. The areas of circles as well as their color intensity show the absolute value of corresponding correlation coefficients. Uncorrected significance is shown highlighting independent measurements, * $p<0.05$ and ** $p<0.01$. Multiple comparison correction was not performed.
